# Chemical Composition of Essential Oil of Leaves from *Lippia schaueriana* Mart. Collected in the Caatinga Area

**DOI:** 10.3390/molecules23102480

**Published:** 2018-09-27

**Authors:** Ana Valéria Vieira de Souza, Uiliane Soares dos Santos, Jackson Rafael de Sá Carvalho, Bruno Djvan Ramos Barbosa, Kirley Marques Canuto, Tigressa Helena Soares Rodrigues

**Affiliations:** 1 Embrapa Semiarid, 56302-970 Petrolina-PE, Brazil; 2 State University of Feira de Santana (UEFS), 44036900 Feira de Santana, Bahia, Brazil; uilianesoares@hotmail.com (U.S.d.S); rafael_.carvalho@hotmail.com (J.R.d.S.C.); brunodj31@hotmail.com (B.D.R.B.); 3 Embrapa Tropical Agroindustry, 60511-110 Fortaleza-CE, Brazil; kirley.canuto@embrapa.br; 4 State Universityof Vale do Acaraú (UVA), 62040-370 Sobral-CE, Brazil; thelenasr@yahoo.com.br

**Keywords:** endemic, medicinal plant, lipia da serra, piperitone, limonene

## Abstract

*Lippia schaueriana* Mart. (Verbenaceae) is an endemic species of Caatinga with a restricted distribution to the states of Bahia and Pernambuco, which presents itself as a potential source of raw material for extraction of essential oil and exploitation by the chemical and pharmaceutical industries. Considering that there are no reports in the literature of research carried out with this species, this paper aimed to establish—for the first time—the chemical composition of its essential oil. The essential oil of the dry leaves at room temperature was obtained by hydrodistillation after 3 h of extraction and the phytochemical analyzes were done by gas chromatography coupled to mass spectrometry (GC/MS). The main compounds found in the oil of leaves were piperitone oxide (51.25%), caryophyllene (17.76%), limonene (8.06%), spathulenol (6.63%), and piperitone (2.90%). The piperitone oxide is a compound described in the literature that shows antinociceptive, cardiovascular, analgesic, and relaxing activities, as well as fungicidal and insecticidal effect, which gives it an interesting potential for the alternative control of agricultural pests.

## 1. Introduction

The genus *Lippia* (Verbenaceae) includes approximately 200 species of herbs, shrubs, and small trees, most of which are distributed in the Neotropical region [1]. Brazil is the center of diversity of the genus, containing 98 species with a high degree of endemism, more than half of which is located in the Serra do Espinhaço in the state of Minas Gerais [2].

*Lippia shaueriana* Mart. is a Caatinga endemic species, whose distribution is restricted to the states of Bahia and Pernambuco. It was described for the first time growing in pastures in Pernambucano state, between Bom Jardim and Cruz de Valerio cities [3]. The plant has a bush structure, with thick and tetragonal branches. It presents oval, rough, opposing leaves with irregular borders [4,5]. In the region, it is popularly known as “lipia da serra”, “alecrim da serra”, and “alecrim de mocó”. The popular denomination “lipia da serra” refers to the place where the species occurs, which can be observed in small hills with altitudes above 400 m. The popular names “alecrim da serra” and “alecrim de mocó” make reference to the bushy rosemary or wild rosemary, as is known the *Lippia gracilis* species. The name mocó comes from the small rodent *Kerodon rupestris* (Caviidae) that occurs in the same region [6].

Although there are no reports in the literature of scientific research conducted with this species to prove any therapeutic effect, its leaves contain essential oils and are used in rural communities in both Pernambuco and Bahia states for inhalations and baths to treat cold, flu, and headaches.

The essential oils are defined by the International Standards Organization (ISO) as the products obtained from parts of plants by steam distillation as well as products obtained by crushing citrus fruit pericarp. They are complex mixtures of volatile substances, lipophilic, liquid, colorless or slightly yellowish, which have as basic characteristic the smell and the flavor. Their constituents vary from terpene hydrocarbons, aldehydes, ketones, phenols, lactones, coumarins, organic acids among others and, in the mixture, there is a variation in the concentration of these compounds, one of them being the majority compound. They are localized in specialized secretory structures, such as glandular, differentiated parenchyma cells, oil channels or even lysigenic or schizoligene bags, and may occur in only one plant organ or in the whole plant [7,8]. 

The essential oils concentration in the different parts of the plants varies qualitatively and quantitatively in relation to several factors, mainly the soil, the climate, the period of the day and the seasons of the year and the types and doses of fertilization [9]. Essential oils obtained in different organs of the same plant can have significantly different chemical composition, odor and physicochemical characteristics [10,11].

Essential oils have extremely important biological functions in the plant kingdom, such as the attraction of pollinating agents and the protection against certain plant pathogens. Due to the pleasant aroma and chemical constitution, which confers significant therapeutic properties, this important metabolite has been a constant target of the chemical, pharmaceutical, and fragrance industries.

The results presented in this study have not been published before and were obtained through the research carried out at Embrapa semi-arid located in Petrolina-PE, Brazil. These results revealed the strong potential of the *Lippia schaueriana* Mart. species as a source of raw material to obtain essential oil that can be used commercially by chemical, pharmaceutical, and fragrance industries. Considering the high potential of this plant species for commercial exploration, as well as the lack of published data, this study aimed, for the first time, to characterize the chemical composition of the essential oil extracted from *L. schaueriana* leaves.

## 2. Results

The results of the essential oil analysis of *L. schaueriana* are presented in Table 1, according to their retention indexes. The chromatogram of oil exhibited 21 peaks, which led to the identification of 19 components, representing 97.99% of the total oil composition. The main compounds found in the oil of leaves were piperitone oxide (51.25%), caryophyllene (17.76%), limonene (8.06%), spathulenol (6.63%), and piperitone (2.90%) (Table 1). 

## 3. Discussion

Piperitone oxide or piperitone are monoterpenes that have been described as major components of the essential oils of species of the genus *Hyptis* spp., *Mentha* spp. and *Clinopodium odorum* [12,13,14]. This first monoterpene is a clear, slightly yellowish liquid, chemically termed 3-isopropyl-6-methyl-7-oxa-bicyclo [4.1.0] heptan-2-one of molecular formula C_10_H_14_O_2_ and molecular weight 166.21. It is soluble in water and organic solvent. Piperitone is derived from the metabolic pathway for the formation of piperitenone oxide, in which cis-pulegone is also derived [15,16] reported that this terpene has three organic functions in its chemical structure and can be used for the synthesis of other compounds.

The essential oil of *Mentha villosa* presents as major compounds rotundifolone and piperitone oxide [16,17]. The latter compound was attributed to analgesic and relaxing activities [18,19], insecticide [16], and antinociceptive [20], beyond the control of protozoan intestinal parasites [21].

In a study of *Mentha rotundifolia*, the concentration of piperitone oxide in the essential oil reached 80% [15] and was used to define *Mentha pulegium* chemotypes [10]. Among several peppermint chemotypes, some also present piperitone oxide as the major compound of essential oil [22,23]. These latter authors attribute that this compound is potentially responsible for cardiovascular effects, antibacterial and antifungal agents, repellents, and retarders of malaria vector reproduction (*Anopheles stephensi*).

Concentrations between 0.1 and 10 ug·mL^−1^ of piperitenone oxide present in some species of *Mentha*, presented a potentiating effect of the contraction induced by acetylcholine (ACh) and doses greater than 30 ug·mL^−1^, were revealedas depressor intestinal smooth muscle in the contractions caused by KCl, Ach, and tetraethylammonium [19].

In addition to the effect on the control of protozoan intestinal parasites, it is also attributed to the relevant piperitenone oxide antimicrobial activity, fungicide, and insecticide, which gives it an interesting potential for alternative pest control [11,15,21].

The chemical composition and relevant antimicrobial activities of other species of the genus *Lippia* spp. have been well investigated and some studies have already shown antifungal and antibacterial activity against several microorganisms of agricultural, medical, and veterinary importance [24,25,26,27,28,29]. However, the compounds reported as effective in this context by the various authors are carvacrol and thymol, the latter being also present in the essential oil of *L. schaueriana* (Table 1). The variation between the chemical composition of the essential oil of *L. schaueriana* and other species of the genus are related to the factors that influence the content of the secondary metabolites in plants, as presented previously [9].

Although this species presents itself as a potential source of raw material for the exploration of industries interested in obtaining products from the Brazilian flora, be it drugs or products with biopesticidal action, there are no records in the literature of any research done with the same, mainly in the field of natural product chemistry.

In this context and considering the endemism of the species with relevant potential of use, the elucidation of the chemical composition of its essential oil represents a significant contribution to the scientific community, since this research can serve as a subsidy for several areas of inter and multidisciplinary science. From these results, chemical, agronomic and pharmaceutical studies can be carried out, in which the sustainable management of the species and of this important Caatinga ecosystem should be sought.

## 4. Materials and Methods

Fresh leaves of *Lippia schaueriana* Mart. were collected in august of 2014 in Petrolina (Coordinates: 9°4′4.19″ S; 40°19′42.24″ W), State of Pernambuco, Brazil. A voucher specimen (HTSA7231) was deposited at the Herbarium of the Semi-Arid Tropic (HTSA) of the Brazilian Agricultural Research Corporation (EMBRAPA). The leaves were dried in room temperature for four days. The dried leaves (100 g) at room temperature was subjected to hydrodistillation for 3 h in a modified Clevenger-type apparatus. The oil yield was found to be 1.2 mL. The oil was dried over anhydrous sodium sulfate. The essential oil obtained had a yellow color and characteristic odor. The oil was stored in a refrigerator until the analysis by GC-MS. 

The compositional analysis the *L. schaueriana* essential oil was investigated in a semi quantitative way on a Shimadzu QP-2010 Gas Chromatograph interfaced to a mass spectrometer (GC-MS). The following conditions were used: DB-5MS column Agilent Technologies (30 m. 0.25 mm. 0.25 μm); helium (99.999%) carrier gas at a constant flow of 1.1 mL/min; 1.0 μL injection volume; injector split ratio of 1:10; injector temperature 250 °C; electron impact mode at 70 eV; ion-source temperature 280 °C and transfer line temperature 260 °C. The oven temperature was programmed from 60 °C to 240 °C at a temperature ramp of 3 °C/min. 

A mixture of linear hydrocarbons (C9H20-C21H40) was injected under the same experimental conditions that the samples and the identification of the constituents was performed by comparing the mass spectra obtained with those of the equipment database (Wiley 7 lib and NIST 08 lib), using the Kovats Index. The data were processed with help of the Shimadzu GC-MS Solution software.

The identification of the components was performed based on comparison of retention indices in literature [30]. For the retention index, the equation of van den Dool and Kratz [31] was used relative to a homologous series of n-alkanes (nC_9_-nC_18_). Three libraries of the equipment WILEY8, NIST107, and NIST21 were used, which allows data comparison of the spectra with those contained in the libraries using an 80% similarity index.

## 5. Conclusions

The results presented in this first study for *Lippia schaueriana* are unpublished. The chemical composition of the essential oil showed the strong potential of this species for commercial use of the chemical, pharmaceutical, and fragrance industries.

This communication reports interesting information about the essential oil of this species and may be the starting point for future work on the biotechnological properties of a species not yet researched scientifically.

## Figures and Tables

**Table 1:** Chemical composition of essential oil of dried leaves of *Lippia schaueriana* (Verbenaceae).

Peak	RT (min)	RI *	RI literature **	Compound	% GC-MS
1	5.79	1025	1024	*p*-Cymene	0.23
2	5.91	1030	1029	Limonene	8.06
3	6.23	1058	1059	*cis*-β Ocimene	0.54
4	7.78	1100	1096	Linalool	0.30
5	8.60	1121	1122	Mentha-2.8-dien-1-ol (trans)	1.05
6	9.06	1135	1137	Mentha-2.8-dien-1-ol (cis)	0.23
7	11.72	1148	1149	Myrcenone	2.04
8	11.78	1208	1229	Trans-carveol	0.53
9	12.66	1236	1243	Carvone	0.55
10	14.13	1291	1290	Thymol	1.10
11	14.28	1299	1299	Carvacrol	0.40
12	15.52	1338	1342	Trans-carvenyl-acetate	1.22
13	15.83	1342	1343	Piperitone	2.90
14	16.56	1370	1368	Piperitone oxide	51.25
15	17.72	1422	1419	β-Caryophyllene	17.76
16	20.81	1498	1500	Bicyclogermacrene	1.59
17	21.05	1509	1505	β-Bysabolene	0.44
18	23.26	1580	1578	Spathulenol	6.63
19	24.86	1585	1583	Caryophyllene oxide	1.77
20	30.15	1865	---	NI	1.14
21	33.63	2127	---	NI	0.29
**Total**					**97.99%**

RT: retention time. * RI: retention indices on DB-5MS column (relative to *n*-alkanes). ** Adams (1995). (---): Not determined. NI: not identified compound.
